# Effect of Hydroponically Grown *Red Panax Ginseng* on Perceived Stress Level, Emotional Processing, and Cognitive Functions in Moderately Stressed Adults: A Randomized, Double-Blind, Placebo-Controlled Study

**DOI:** 10.3390/nu17060955

**Published:** 2025-03-09

**Authors:** Valérie Dormal, Lucas Jonniaux, Marine Buchet, Laurent Simar, Sylvie Copine, Louise Deldicque

**Affiliations:** 1 Center of Investigation in Clinical Nutrition, Université Catholique de Louvain, 1348 Louvain-la-Neuve, Belgium; lucas.jonniaux1@gmail.com (L.J.); marine.buchet@uclouvain.be (M.B.); docteur.simar@outlook.com (L.S.); sylvie.copine@uclouvain.be (S.C.); louise.deldicque@uclouvain.be (L.D.); 2 Institute of Neuroscience, Université Catholique de Louvain, 1348 Louvain-la-Neuve, Belgium

**Keywords:** *Red Panax ginseng* root powder, stress, cognitive function, depression, clinical trial

## Abstract

**Background/objectives:** Chronic stress is a pervasive issue affecting individuals worldwide, with profound implications for mental and physical well-being. Panax ginseng, a widely used herbal supplement renowned for its adaptogenic properties, is hypothesized to alleviate some stress effects. This study aims to evaluate the impact of hydroponically grown *Red Panax ginseng* root powder with a high level of rare ginsenosides supplementation on perceived stress levels, as well as on the emotional and cognitive abilities of moderately stressed participants. **Methods:** A randomized, double-blind, controlled study was conducted with 149 participants. They were randomly assigned to either the Ginseng supplementation group (N = 72; 200 mg/day, including 24 mg of ginsenosides) or the Placebo group (N = 77). The intervention lasted for 3 weeks. The perceived stress level was measured at baseline (D0) and at the end of the intervention (D21) using a validated scale (PSS) alongside assessments of emotional (BDI and PANAS) and cognitive abilities (CANTAB subtests). **Results:** Significantly larger decreases in the PSS and negative affect score (PANAS) were observed following intervention in the Ginseng group compared with the Placebo group. Compared to the Placebo group, participants in the Ginseng group showed faster response latencies during a spatial planning task (OTSC subtest). In addition, there was a marginally larger decrease in the BDI score in the Ginseng group. **Conclusions:** These results confirm the emotional and cognitive benefits of *Red Panax ginseng* in moderately stressed adults and pave the way for further exploration of its use as a promising approach to improving psychological well-being.

## 1. Introduction

Chronic stress has emerged as a significant public health concern, with detrimental effects on both mental and physical well-being. Perceived stress, defined as the subjective appraisal of stressors exceeding an individual’s ability to cope, plays a pivotal role in the development and exacerbation of various health conditions, including anxiety, depression, cardiovascular diseases, and impaired cognitive function [1,2]. As its levels continue to rise within society, there is a growing momentum in the pursuit of effective interventions to alleviate the adverse effects of stress.

One of those avenues of exploration is the use of dietary and botanical supplements, in particular, *Panax ginseng C.A. Mey.*, a widely used herbal remedy in traditional Chinese medicine [3,4,5]. Renowned for its adaptogenic properties, *Panax ginseng* has attracted attention for its supposed ability to modulate the body’s response to stress factors, thereby promoting resilience and improving overall health [4,5,6,7].

*Panax ginseng*, also known as Asian ginseng or Korean ginseng, belongs to the Araliaceae family and is rich in bioactive compounds such as ginsenosides, polysaccharides, and polyphenols, which are believed to confer its pharmacological effects [6]. Those compounds have multifaceted effects on various physiological systems, ranging from regulating neurotransmitter activity to modulating stress response pathways [6,8]. While traditional ginsenosides are known to offer multiple health benefits, emerging research highlights the unique potential of rare ginsenosides [9,10]. Unlike classic ginsenosides, which are abundant in raw ginseng and act as precursors, rare ginsenosides are formed through specific processing methods, such as steaming and fermentation. These methods enhance their bioavailability and potency, allowing them to exert more pronounced neuroprotective, anti-inflammatory, and antioxidative effects. Consequently, these rare ginsenosides are more effective in managing stress-induced symptoms and improving cognitive functions, as they bypass the gut-mediated conversion process that limits the efficiency and speed of action of traditional ginsenosides [11].

Preclinical studies have provided valuable information on the mechanisms underlying the stress-modulating properties of *Panax ginseng*. Those mechanisms include the modulation of the hypothalamic–pituitary–adrenal (HPA) axis, regulation of neurotransmitter systems (serotonin, dopamine, gamma–aminobutyric acid), attenuation of oxidative stress and inflammation, and enhancement of neuroplasticity and neuroprotection within key brain regions implicated in stress regulation [12,13].

In human clinical trials, *Panax ginseng* has demonstrated promising effects in mitigating stress-related symptoms. Several studies have reported improvements in perceived stress levels, mood regulation, and cognitive function following ginseng supplementation. For instance, randomized, double-blind, placebo-controlled trials have shown that ginseng supplementation can lead to reductions in self-reported stress levels and improvements in overall well-being either after one single dose [14,15] or 4-week intervention [7]. Moreover, ginseng has been associated with enhancements in cognitive performance, including attention, memory, and executive function, in tired, healthy subjects [14] or individuals exposed to high stress levels [16]. These findings suggest that *Panax ginseng* may exert beneficial effects on stress resilience and psychological well-being in human populations, highlighting its potential as a complementary therapeutic approach for stress management. However, there are notable limitations to consider, including small sample sizes, variability in doses administered, and intervention durations, as well as the lack of clarity regarding the nature of the products used, whether standardized extracts, root powder, or other forms. Furthermore, the levels of active compounds, such as classic or rare ginsenosides, are not always specified, which can significantly influence the results. This heterogeneity complicates the interpretation of findings and prevents the establishment of conclusive results. Additionally, subjective stress assessment introduces potential biases, as individual perceptions of stress can differ. Moreover, the long-term safety and efficacy of *Panax ginseng* supplementation remain unclear [17,18], requiring further research.

Therefore, the aim of this randomized, double-blind, placebo-controlled study was to test the effect of a 3-week intervention of a unique formulation of hydroponically grown *Red Panax ginseng* root powder enriched with high levels of rare ginsenosides (200 mg/day) on a large sample of adult participants experiencing moderate degree of stress. The effect was evaluated using a validated scale on the level of perceived stress as well as on their emotional abilities and cognitive function.

## 2. Materials and Methods

### 2.1. The Red Panax Ginseng Root Powder

#### 2.1.1. Cultivation Cycle

The *Panax ginseng* roots were hydroponically cultivated using an innovative vertical farming technology, according to FSSCC22000 certified standards, ensuring the strict control and stability of growing conditions and, therefore, reproducible chemical composition and high purity. Korean-origin seeds have been cultivated in Europe for many years. Building on this genetic foundation, the company has developed its own cultivar while ensuring the genetic stability of the plant. The innovative vertical farming technology used is a root-exclusive cultivation technique, which eliminates any impact from seed quality. The roots are initially obtained through a research-based process and are then multiplied under controlled conditions. Since this cultivation method is exclusively focused on root growth, photoperiod is not required.

The roots are placed in a Murashige and Skoog culture medium containing a sufficient carbon source, sucrose, to support optimal development. Additionally, a balanced supply of macronutrients, micronutrients, and vitamins is provided to ensure proper growth. The Murashige and Skoog (MS) medium is widely used for in vitro plant culture, including ginseng. Studies have shown its effectiveness in supporting adventitious root growth [19,20] and shoot regeneration without exogenous hormones [21], making it a versatile tool for optimizing metabolite production in plant biotechnology.

Each cultivation cycle lasts 27 weeks, with the culture maintained at a constant temperature of 20 °C, ensuring stable and controlled growing conditions. Harvested ginseng roots were then air-dried and steam-cooked (during two successive processes, each lasting 90 min at 130 °C) to obtain red ginseng roots. Then, the red ginseng was powdered and sifted at 300 µm.

#### 2.1.2. Compound Extraction, Chemicals, and Reference Standards

Compound extraction and analysis were performed as follows. The *Red Panax ginseng* root powder (1 g) was extracted in 100 mL of 70% methanol at 80 °C under agitation for >8 h. The extract solution was filtrated through a 0.45 mm Millipore filter and used for the UHPLC analysis. The reagents used in this study include methanol (≥99.8%), obtained from HiPerSolv CHROMANORM^®^ (VWR Chemicals, Leuven, Belgium; Ref. 20864.360), prepared by mixing 1400 mL of gradient-grade methanol with 600 mL of osmotic water in a 2000 mL flask. Acetonitrile (≥99.9%) was purchased from HiPerSolv CHROMANORM^®^ (VWR Chemicals, Leuven, Belgium; Ref. 20030.360), and phosphoric acid (85%) was sourced from Sigma-Aldrich (Hoeilaart, Belgium; Ref. 345245-100 ML). Water used for analysis was of HPLC grade to ensure high purity and minimal contamination. Additionally, a 10% isopropanol solution, prepared by mixing 10 mL of isopropanol in 100 mL of osmosed water, was used for cleaning the equipment and tubing.

The reference standards used in this study, along with their purity levels, were chosen to ensure the accuracy and reliability of the analytical methods applied: Rg1, Re, Rf, Rb1, Rc, Rh1, Rb2, Gypenoside XVII, Rb3, Ro, F1, Rd, Rg6, F4, F2, Rk3, Rh4, Rg3, Rk1, CK, Rg5, Rh2, Rk2, Rh3, Protopanaxadiol, Protopanaxatriol (purity level ≥ 98%); Rg2, Rb3, Rh2 (purity level ≥ 97%); Rk1, CK, Rk2 (purity level ≥ 95%); Rh1, Rg6, Rg5, Rh3 (purity level ≥ 90%).

Ginsenosides contents (total and rare) were quantified on SHIMADZU UHPLC LC20 ADXR. The UHPLC method used in this study was initially developed and validated at Celabor, a scientific and technical service center. It was later adapted for a Shimadzu UHPLC system in Shimadzu laboratories to ensure compatibility, robustness, and optimal analytical performance. It is a modular system that consists of an SPD-40V Detector, SIL-40C Autosampler, LC-40B XR Pump, and Column oven CTO-40C with column Shim-pack GIST C18 2 µm (150 × 2.1 mm). The sample injection volume was set at 10 µL, analysis was performed at 40 °C, and the detection wavelength was 192 nm. Separation was achieved by elution using a linear gradient with solvent A (0.1% phosphoric acid solution) and solvent B (acetonitrile). The gradient was as follows: t = 0 min, 80% A; t = 40 min, 10% A; t = 45 min, 10% A; t = 46 min, 80% A. The flow rate was set at 0.25 mL/min. All in all, 23 ginsenosides are analyzed. Standards of ginsenosides were purchased (Rh1, Rb3, F1, Rd, Rg6, F2, Rh4, Rg3, Rk1, Rg5, Rh3, and 20S-protopanaxtriol from Sigma Aldrich (Hoeilaart, Belgium); Rg1, Re, Rf, Rb1, Rc, Rb2, and Rh2 from Extra synthese (Genay, France); F4, Rk2, and Rk3 from Chemfaces (Wuhan, China); protopanaxdiol and 20R-protopanaxtriol from VWR (Leuven, Belgium)), and calibration curves were created. As part of monitoring and quality control, calibration was performed every three months. For this purpose, a standard solution was prepared by dissolving 10 mg of standard in 100 mL of 70% methanol (MeOH 70%), resulting in a final concentration of 100 ppm. To ensure measurement accuracy, three UHPLC analyses were performed for each standard with variable injection volumes, selected to ensure that the measured ginsenoside concentrations fell within the range defined by the calibration curve. The method was evaluated in terms of linearity through a calibration curve built over multiple concentration levels. For each standard, linearity was verified to ensure consistency and accuracy.

#### 2.1.3. Study Preparations

One Red ginseng capsule was standardized for the content of 200 mg powdered *Panax ginseng* Meyer root powder, corresponding to 22.4 mg of ginsenosides and 20.2 mg of rare ginsenosides. The placebo capsule contained 200 mg of rice flour and brown sugar. Herbal preparation quality was tested by HPLC in accordance with specifications using appropriate reference standards.

Study preparations were packed and labeled as per national requirements regarding their use for clinical trial investigations. The label included the drug name, study code, and storage conditions.

### 2.2. Participants

**Figure 1:**
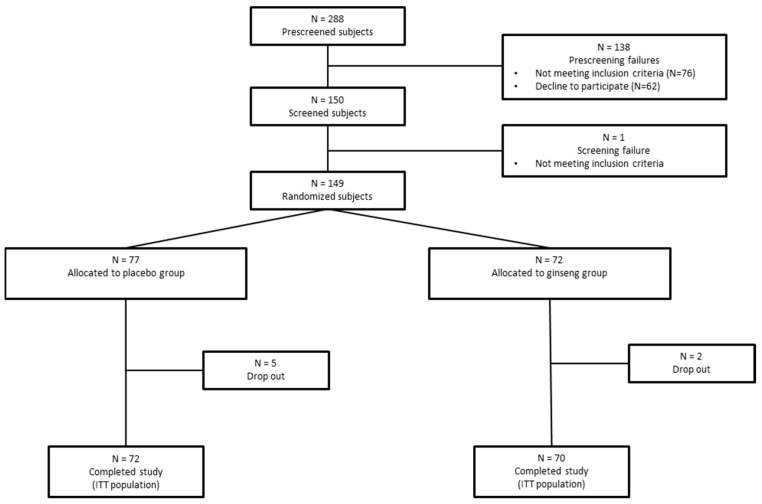
Flow chart of the study. Flow chart of the study samples, including the number of participants who were screened, underwent randomization, completed the study treatment, and were analyzed for the primary and secondary outcomes. ITT, intention-to-treat.

A total of 150 participants were assessed for eligibility, and 149 participants were randomized between June 2023 and December 2023 into the Ginseng (*n* = 72) or Placebo (*n =* 77) groups (Figure 1). Seven participants dropped out of the study (5 for personal reasons and 2 for medical reasons; 5 from the Placebo group and 2 from the Ginseng group). Participants were recruited by posters, mail, and social networks. To be included, the participants had to meet the following criteria: woman or man aged between 18 and 60 years; with a moderate level of perceived stress (scores ranging from 14 to 26 on the Perceived Stress Scale; PSS); speaking French; and stated willingness to comply with all study procedures and availability for the duration of the study. The participants were excluded if they presented one of the following exclusion criteria: severe medical or cognitive problems, which could interfere with the evaluation of the study criteria or with participant safety; a coffee consumption of more than 5 cups per day; an alcohol consumption exceeding 3 glasses of wine per day, 2 pints of beer per day, or one glass of strong alcohol per day; current consumption of drugs and/or participants with historical drug addiction (<5 years); participants undergoing medical treatment, which could have interfered with cognitive and emotional processing; type 1 or type 2 diabetes; and woman of childbearing age who was pregnant or breastfeeding or who wished to become pregnant within the next 6 weeks or who was not using an adequate method of contraception (e.g., oral contraception, intra-uterine device, abstinence, etc.).

All selected participants provided written informed consent. The trial was approved by the local ethical committee and was carried out in accordance with the Declaration of Helsinki and the Good Clinical Practice, as required by the following regulations: the Belgian law of 7 May 2004 regarding experiments in human beings and the EU Directive 2001/20/EC on Clinical Trials (registration at clinicaltrials.gov accessed on 14 May 2024 as NCT06414486).

### 2.3. Study Design

This study was a randomized, double-blind, placebo-controlled interventional study. A pre-screening was proposed with an online questionnaire sent by e-mail. Then, the screening visit (D0), comprising a physical examination (including measurement of body weight, height, and heart rate) and an evaluation of baseline perceived stress (using the PSS), was organized. The participants, who all met the criteria, were randomly assigned to the Ginseng or the Placebo groups and invited to take the first capsule. After this, a standardized breakfast was proposed. Then, an evaluation of cognitive and emotional processing was carried out. Participants completed two subtests of the Cambridge Neuropsychological Test Automated Battery (CANTAB) investigating memory (Paired Associate Learning, PAL) and executive functions (One Touch Stockings of Cambridge, OTSC), respectively, and self-completed questionnaires assessing positive and negative affects (Positive and Negative Affect Schedule, PANAS), and the level of depression (Beck’s Depression Inventory, BDI).

During the intervention, both groups were instructed to ingest one capsule with a glass of water every morning before lunch for the next 20 days. The capsule contained 200 mg of *Red Panax ginseng* in the Ginseng group and 150 mg of brown sugar and 50 mg of rice flour in the Placebo group. The capsules used were transparent, and the contents were of a similar color, making identification impossible.

The second visit (D21) was scheduled 3 weeks after visit 1 (D0). During this visit, concomitant medication and adverse events were recorded. Perceived stress levels and cognitive and emotional processing were also evaluated. Participants gave back unused capsules for the evaluation of compliance.

Safety and tolerability of the *Red Panax ginseng* were defined as the occurrence of adverse events. All events were recorded from the signature of the informed consent to the end of the study. Compliance was assessed at the end of the study by comparing the remaining quantity of capsules to the quantity given at the beginning of the study.

### 2.4. Emotional and Cognitive Processing Measures

The perceived stress level was evaluated by the PSS [22], which contains 10 questions with responses measured using a 5-point Likert scale. The severity of depression was assessed by the BDI [23], which is a 21-question multiple-choice self-report inventory. All items are scored on a 4-point scale, ranging from 0 to 3. Emotional processing was evaluated by the PANAS [24], which is a self-report scale questionnaire that consists of different words that describe feelings and emotions. In the PANAS questionnaire, 10 items evaluate the positive effect, whereas 10 items evaluate the negative effect.

Finally, two subtests of the CANTAB [25] were used to investigate cognitive processing. The PAL subtest assessed visual memory and new learning. In this subtest, boxes are displayed on the screen and are “opened” in a randomized order. One or more of them contain a pattern. The patterns are then displayed in the middle of the screen, one at a time, and the participant must select the box in which the pattern was originally located. If the participant makes an error, the boxes are opened in sequence again to remind the participant of the locations of the patterns. Outcome measures include the total errors (adjusted) and first-attempt memory score. The OTSC subtest assessed both the spatial planning and the working memory subdomains. The participant is shown two displays containing three colored balls. The displays are presented in such a way that they can be easily perceived as stacks of colored balls held in stockings or socks suspended from a beam. There is a row of numbered boxes along the bottom of the screen. The test administrator first demonstrates to the participant how to move the balls in the lower display to copy the pattern in the upper display and completes one demonstration problem, where the solution requires one move. The participant must then complete three further problems, one each requiring two moves, three moves, and four moves. Next, the participant is shown further problems and must work out in their head how many moves the solutions require; then, they select the appropriate box at the bottom of the screen to indicate their response. Outcome measures include the mean latency to the first choice and the number of problems solved on the first choice.

### 2.5. Statistical Analyses

The sample size was calculated based on the primary endpoint, i.e., changes in the perceived stress level assessed by the PSS questionnaire. Using PASS 14.0.7 software and the one-sided two-sample *t*-test, 75 subjects per group (71 + 5% dropout) were needed to observe a decrease of 1.2 points in the PSS in the Ginseng group compared to the Placebo. The standard deviation was fixed at 2.2, power 80%, and alpha at 0.05.

Statistical analyses were performed using the software systems SPSS version 24 software (IBM SPSS Statistics, Chicago, IL, USA). Baseline differences in demographic characteristics between the groups were examined using χ^2^ tests (for categorical variables) and independent *t*-tests (for continuous variables). As variables were not normally distributed, the evolution of an endpoint between the baseline and day 21 within a group was evaluated using the non-parametric Wilcoxon signed-rank test. This test can determine the intervention effect inside a group. The difference from baseline after 21 days was compared between groups using the non-parametric Mann–Whitney two-sample test. This test determines the intervention effect between groups, considering the baseline. Data are shown as the mean with SD. All analyses were conducted at an alpha level of 0.05.

## 3. Results

### 3.1. Composition of the Red Panax Ginseng Root Powder

**Figure 2:**
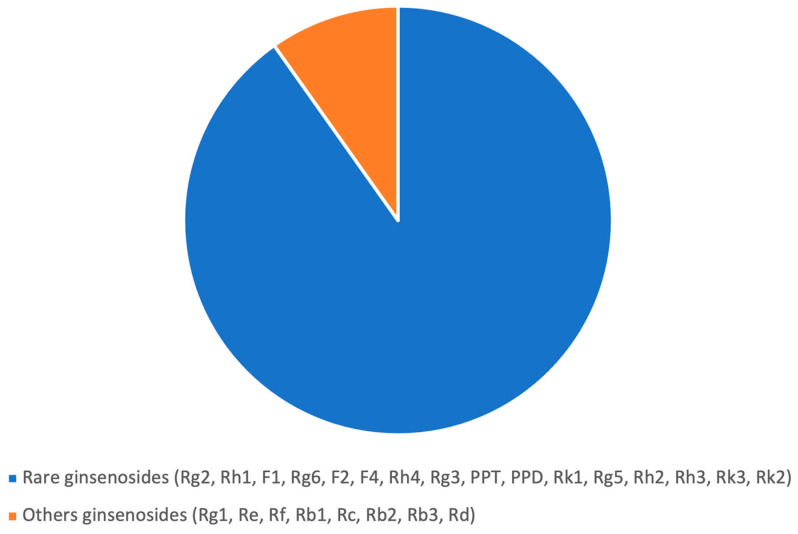
Analysis of ginsenosides content of the *Red Panax ginseng* root powder by UHPLC.

The analysis of the ginsenosides content of the *Red Panax ginseng* root powder by UHPLC revealed a high level of ginsenosides (11.2%; i.e., 11.2 g of ginsenosides per 100 g of dry root powder). Moreover, the part of rare ginsenosides was very elevated (90.2%; Figure 2). The ginsenoside profile of this preparation is similar to the ginseng cultivated in the field, but the ginsenoside content is two- to three-fold higher.

**Figure 3:**
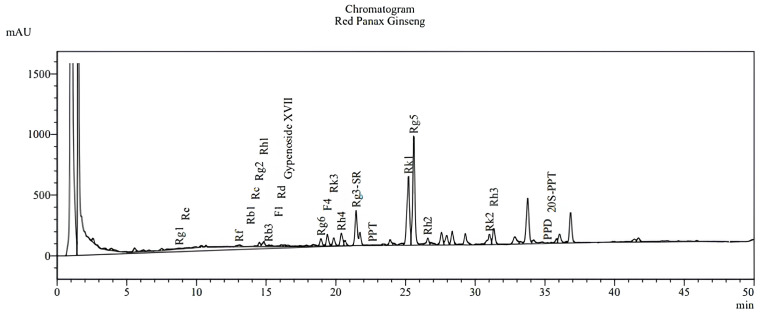
Chromatograms of the performed analyses of the *Red Panax ginseng* root powder.

The results, including the retention times of the analyzed compounds (Table 1) and chromatograms of the performed analyses (Figure 3), are presented below.

### 3.2. Baseline Evaluation

The two groups showed no baseline differences in age, gender, body mass index (BMI), heart rate, and PSS score (Table 2).

### 3.3. Stress and Emotional Abilities

**Figure 4:**
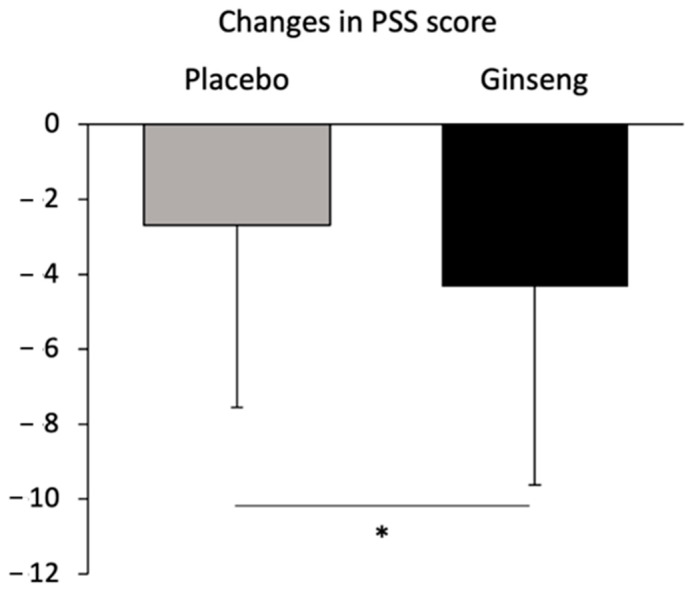
Changes in the PSS score. Changes in the PSS score from baseline (D0) to D21 in the Placebo and Ginseng groups. Data are presented as mean ± SD. * *p* < 0.05.

The descriptive statistics for the stress and emotional scores are presented in Table 3. After 21 days of intervention, an improvement in the PSS score was observed in both groups (Placebo = −2.7 ± 4.7, *p* < 0.001; Ginseng = −4.3 ± 4.6, *p* < 0.001), with a larger improvement in the Ginseng vs. the Placebo group (*p* = 0.040; Figure 4).

The BDI score decreased in both the Placebo and Ginseng groups after 3 weeks of intervention compared to baseline (−4.7 ± 6.8, *p* < 0.001, and −6.4 ± 6.4, *p* < 0.001, respectively). The BDI score change from D0 to D21 tended to be larger in the Ginseng vs. the Placebo group (*p* = 0.073).

Regarding emotional processing, the PANAS Negative score decreased only in the Ginseng group after the intervention (−2.6 ± 5.9, *p* < 0.001), resulting in a different evolution from D0 to D21 between the Ginseng and the Placebo group (*p* = 0.032). No difference in the PANAS Positive score was observed at D0 and at D21 for either group.

### 3.4. Cognitive Processing

The descriptive statistics for the PAL and OTSC subtests are presented in Table 4. After 21 days of intervention, a decrease in the total errors of the PAL subtest was observed in the Ginseng group only (−2.2 ± 7.4, *p* = 0.029). The first attempt memory score increased from D0 to D21 in both groups (Ginseng = +0.8 ± 2.8, *p* = 0.006; Placebo = +1.2 ± 3.4, *p* = 0.050). No difference between the Ginseng and Placebo groups was measured in the evolution of the total errors and the first attempt memory score from D0 to D21.

After 21 days of intervention, a shortening in the median latency to first choice during the OTSC subtest was observed only in the Ginseng group (−1633 ± 4304 ms, *p* = 0.002), resulting in a different evolution from D0 to D21 between the Ginseng and the Placebo group (*p* = 0.036). The number of problems solved on the first choice was not different between groups at D0 and D21.

### 3.5. Safety and Compliance

The Ginseng supplementation was well tolerated, with no serious adverse event reported and no difference in the mild and moderate adverse events reported by the Placebo (*n =* 11) and Ginseng (*n =* 21) groups. Only four moderate adverse events were reported (three in the Ginseng group and one in the Placebo group) and were not related to the study product (i.e., traffic accident, flu, and migraines). A high compliance was observed with an intake of 98.4 ± 3.8% in the Ginseng group and 97.9 ± 3.6% in the Placebo group.

## 4. Discussion

The aim of this randomized, double-blind, placebo-controlled study was to evaluate the impact of hydroponically grown *Red Panax ginseng* root powder with a high level of rare ginsenosides supplementation (200 mg/day for 3 weeks) on perceived stress level, as well as on the emotional and cognitive abilities of moderately stressed participants.

The originality and the added value of the present study was to use a hydroponically grown *Red Panax ginseng* root power of very high quality. The key active constituents of *Panax ginseng* are ginsenosides, but not all ginsenosides are equally bioactive. The most abundant ginsenosides of commercial ginseng are Rb1, Rb2, Rc, Rd, Re, Rf, and Rg1, and they are poorly absorbed after oral ingestion [11]. These ginsenosides undergo deglycosylation by colonic bacteria to intestinal metabolites. The metabolites are the primary compounds that appear in systemic circulation after oral administration of ginsenosides; they are also widely accepted as being responsible for the pharmacologic activity of ginseng [11]. Rare ginsenosides, less abundant in ginseng, are ginsenosides metabolites and the most pharmacologically active ginsenosides. The transformation of ginsenosides into rare ginsenosides by changing the chemical structure of ginsenosides can occur through physical methods (steaming), chemical methods (acid or alkaline hydrolysis), and biotransformation (bacterial/fungal fermentation) [26]. Here, red ginseng, i.e., steamed ginseng, which increases the concentration of rare ginsenosides, was developed to enhance the oral absorption of *Panax ginseng* components and increase its pharmacologic efficacy.

Using this specifically developed *Red Panax ginseng* root powder, meaningful insights have been gained from our study at different levels. Firstly, the observed larger reduction in perceived stress levels among participants in the Ginseng group compared to the Placebo group underscores the potential therapeutic efficacy of *Red Panax ginseng* in mitigating stress. These results extend those already observed in clinical studies in human participants where a single dose was administered [14,15] or during a 12-day [14] or 4-week [16] intervention. Here, we confirm similar effects on a large sample of adult participants (*n =* 149) with a dosage of *Red Panax ginseng* of 200 mg per day (i.e., 22.4 mg of active substance). In most previous studies, a higher dose (400 mg/day or 2 g/day) was required to demonstrate a significant effect on stress, e.g., [14,15]. It is worth noting that the use of the PSS, a validated and frequently used measure [27], provided a standardized and reliable evaluation, facilitating the inclusion of our participants in the study as well as the comprehensive assessment and comparison of stress levels within our sample. Importantly, this study also demonstrated that, at this dosage and during the 3-week intervention, *Red Panax ginseng* was safe and well-tolerated, with very few adverse events possibly related to the product reported by the participants. However, the long-term effects of the regular intake of *Panax ginseng* are still unknown and require further investigation.

Secondly, our study revealed a larger reduction in negative effects, as measured by the negative part of the PANAS, within the Ginseng group compared to the Placebo group. We also observed a downward trend for the BDI score, reflecting the level of perceived depression, following the intervention in the Ginseng group. Together, these results suggest that *Red Panax ginseng* supplementation may contribute to improving emotional well-being by decreasing negative emotional states. This finding is particularly relevant as negative affect is closely linked to stress and psychological distress [28].

Finally, the observed shorter response latencies in the OTSC subtest of the CANTAB, specifically evaluating spatial planning abilities, among participants receiving *Red Panax ginseng* supplementation highlights potential cognitive enhancement effects. Moreover, participants in the Ginseng group made fewer errors in a visual memory learning task (PAL subtest) after the intervention. While some studies failed to demonstrate the beneficial effects of red ginseng on performance in cognitive tasks measuring attentional abilities and concentration [29,30], others were able to detect improvements in working memory [29,31], long-term visual memory tasks [30,32], or executive functions [32], which we partially confirmed here on a very high number of participants. Altogether, those results suggest that *Red Panax ginseng* may exert beneficial effects on certain cognitive functions, particularly those related to spatial planning abilities and visual memory.

## 5. Conclusions

Overall, our study provides compelling evidence supporting the therapeutic potential of hydroponically grown *Red Panax ginseng* root powder with high levels of rare ginsenosides in ameliorating stress levels, enhancing emotional well-being, and improving cognitive functions for individuals experiencing moderate levels of stress. These findings underscore the importance of further research to explore the long-term implications of *Red Panax ginseng* supplementation in clinical populations, possibly presenting higher levels of stress and/or anxiety, as well as depressive feelings. The beneficial effects observed remain moderate; it is important to consider *Red Panax ginseng* supplementation as a complementary therapeutic contribution to a more comprehensive treatment.

## Figures and Tables

**Table 1:** Retention times and concentrations of the analyzed compounds of the *Red Panax ginseng* root powder.

Name	Ret. Time	Conc. (mg/g of DM)
Rg1	8758	0.31
Re	9208	0.19
Rf	13,052	0.77
Rb1	13,895	0.18
Rg2	14,503	0.79
Rc	14,228	0.34
Rh1	14,822	1.09
Rb2	--	--
Rb3	15,182	0.69
Ro	--	--
F1	15,884	0.16
Rd	16,092	0.66
Gypenoside XVII	16,556	2.04
Rg6	18,927	1.92
F4	19,394	4.33
F2	--	--
Rk3	19,843	1.92
Rh4	20,393	19.05
Rg3-SR	21,449	16.03
PPT	22,635	0.10
Rk1	25,214	20.81
C(k)	--	--
Rg5	25,587	13.99
Rh2	26,589	2.33
Rk2	31,023	21.12
Rh3	31,337	3.16
20S-PPT	35,476	0.23
PPD	35,192	0.11

**Table 2:** Baseline subject’s characteristics in the Ginseng and Placebo groups.

Variables	Ginseng Group (*n* = 70)	Placebo Group (*n* = 72)	
	Mean (SD)	Mean (SD)	*p*-Value
Age (y)	29 (10)	31 (12)	0.904
Gender (M)	41%	47%	0.487
BMI (kg/m^2^)	23.6 (4.1)	24.0 (3.9)	0.674
Heart rate (bpm)	70 (15)	69 (11)	0.581
PSS	21.7 (3.6)	20.8 (3.9)	0.187

BMI, body mass index; PSS, perceived stress scale; SD, standard deviation.

**Table 3:** Stress and emotional processing scores in the Ginseng and Placebo groups at baseline (D0), after the intervention (D21), and the difference between D21 and D0.

Variables Mean (SD)	Ginseng Group (*n* = 70)			Placebo Group (*n* = 72)		
	D0	D21	D21−D0	D0	D21	D21−D0
PSS	21.7 (3.6)	17.3 (4.8) *	−4.3 (4.6) ^$^	20.8 (3.9)	18.1 (5.5) *	−2.7 (4.7)
BDI	16.5 (7.9)	10.1 (7.5) **	−6.4 (6.4)	14.3 (8.5)	9.6 (8.2) **	−4.7 (6.8)
PANAS Positive	29.4 (6.5)	28.8 (6.5)	−0.6 (5.7)	29.6 (5.4)	28.8 (6.3)	−0.7 (4.6)
PANAS Negative	19.7 (6.2)	17.1 (6.1) **	−2.6 (5.9) ^$^	17.5 (6.0)	16.5 (5.9)	−1.1 (4.6)

Different from D0 within a group: * *p* < 0.05, ** *p* < 0.001 (Wilcoxon signed-rank test). Different changes from D0 to D21 between groups: ^$^ *p* < 0.05 (Mann–Whitney two-sample test).

**Table 4:** PAL and OTSC subtest outcomes in the Ginseng and Placebo groups at baseline (D0), after the intervention (D21), and the difference between D21 and D0.

Variables Mean (SD)		Ginseng Group (*n* = 70)			Placebo Group (*n* = 72)		
		D0	D21	D21−D0	D0	D21	D21−D0
PAL	Total errors (adjusted)	7.8 (9.0)	5.7 (6.5) *	−2.2 (7.4)	6.0 (8.0)	5.2 (7.5)	−0.8 (6.4)
	First attempt memory score	15.4 (3.6)	16.6 (3.0) *	1.2 (3.4)	16.0 (3.4)	16.8 (3.2) *	0.8 (2.8)
OTSC	Median latency to first choice (ms)	9982 (4297)	8374 (3191) *	−1633 (4304) ^$^	9335 (5058)	8998 (4495)	−374 (3628)
	Number of problems solved on first choice	11.2 (2.4)	11.6 (2.1)	0.4 (2.1)	11.5 (2.1)	11.4 (2.3)	0.0 (2.0)

Different from D0 within a group: * *p* < 0.05 (Wilcoxon signed-rank test). Different changes from D0 to D21 between groups: ^$^ *p* < 0.05 (Mann–Whitney two-sample test).

## Data Availability

Data are available upon request by sending an e-mail to cicn@uclouvain.be.
